# Supplementary material for: Near-term pregnant women in the Dominican Republic experience high rates of Group B Streptococcus rectovaginal colonization with virulent strains
Source: PLOS Glob Public Health. 2023 Sep 21;3(9):e0002281. doi: 10.1371/journal.pgph.0002281 (PMC10513192; doi:10.1371/journal.pgph.0002281)
Supplement: S1 Fig — (DOCX) [file pgph.0002281.s003.docx]

**S1 Figure. Pregnant women assessed for study eligibility.**

**Pregnant women**

**assessed for eligibility**

*n* = 1,365

**Eligible**

*n* = 486

**Enrolled**

*n* = 350

**Inclusion criteria not met:**

Gestational age <35 weeks at recruitment (*n* = 645)

Unable to understand and speak Spanish (*n* = 183)

Age <18 years^1^ (*n* = 51)

Declined to participate^2^ (*n* = 136)

^1^Participants <18 years of age were not eligible for inclusion during the first two months of enrollment due to need for additional approval from the relevant Institutional Review Boards. Approval was subsequently met, and participants of all ages were considered eligible for inclusion for the remainder of the study period.

^2^The majority of women who declined consent reported doing so because they were scheduled for Cesarean section or induction of labor on that day.
