# Supplementary material for: Near-term pregnant women in the Dominican Republic experience high rates of Group B Streptococcus rectovaginal colonization with virulent strains
Source: PLOS Glob Public Health. 2023 Sep 21;3(9):e0002281. doi: 10.1371/journal.pgph.0002281 (PMC10513192; doi:10.1371/journal.pgph.0002281)
Supplement: S1 Table — (DOCX) [file pgph.0002281.s004.docx]

**S1 Table.** Isolate-level data for Group B *Streptococcus* (GBS) strains collected in late pregnancy in the Dominican Republic. Sequence type (ST), clonal complex (CC), serotype, and antimicrobial resistance data were derived from whole-genome sequences. Novel sequence types (2113, 2114, 2115, and 2116) were assigned by PubMLST.

| **Strain** | **ST** | **CC** | **Serotype** | **PBP2x**  **Mutations** | **MLS**  **Resistance** | **Tetracycline**  **Resistance** | **Quinolone**  **Resistance** |
| --- | --- | --- | --- | --- | --- | --- | --- |
| DR3003 | 17 | CC17 | III | I377V; G627V |  | *tetM* |  |
| DR3006 | 144 | CC23 | Ia | I377V; V510I |  | *tetM* |  |
| DR3008 | 1 | CC1 | Ib | None |  | *tetM* |  |
| DR3009 | 28 | CC19 | II | None | *ermB* | *tetO* |  |
| DR3010 | 1010 | CC452 | IV | I377V; V510I |  |  |  |
| DR3011 | 23 | CC23 | Ia | I377V; V510I |  | *tetM* |  |
| DR3014 | 23 | CC23 | Ia | I377V; V510I |  | *tetM* |  |
| DR3015 | 28 | CC19 | II | None |  | *tetM* |  |
| DR3021 | 1010 | CC452 | IV | I377V; V510I; V577L |  |  |  |
| DR3025 | 17 | CC17 | III | G329V; I377V; G627V |  | *tetM* |  |
| DR3027 | 1010 | CC452 | IV | I377V; V510I |  |  |  |
| DR3029 | 144 | CC23 | Ia | I377V; V510I |  | *tetM* |  |
| DR3040 | 1010 | CC452 | IV | I377V; V510I |  |  |  |
| DR3044 | 24 | CC452 | Ia | I377V; V510I |  | *tetM* |  |
| DR3047 | 498 | CC452 | V | I377V; V510I |  | *tetM* |  |
| DR3053 | 1010 | CC452 | IV | I377V; V510I |  |  |  |
| DR3055 | 1010 | CC452 | IV | I377V; V510I |  |  |  |
| DR3060 | 347 | CC19 | II | None | *ermB* | *tetO* |  |
| DR3061 | 1 | CC1 | II | None |  | *tetM* |  |
| DR3063 | 24 | CC452 | V | I377V; V510I |  | *tetM* |  |
| DR3067 | 2113 | No CC | Ia | I377V; V510I |  | *tetM* |  |
| DR3072 | 1010 | CC452 | IV | I377V; V510I |  |  |  |
| DR3075 | 23 | CC23 | Ia | I377V; V510I |  | *tetM* |  |
| DR3079 | 17 | CC17 | III | I377V; G627V |  | *tetM* |  |
| DR3087 | 1010 | CC452 | IV | I377V; V510I |  |  |  |
| DR3088 | 17 | CC17 | III | I377V; G627V |  | *tetM* |  |
| DR3089 | 1 | CC1 | II | None |  | *tetM* |  |
| DR3096 | 23 | CC23 | Ia | I377V; V510I |  | *tetM* |  |
| DR3097 | 19 | CC19 | V | None | *ermB* | *tetM; tetO* | ParC-S79F |
| DR3099 | 1 | CC1 | V | None | *ermTR* | *tetM* |  |
| DR3102 | 19 | CC19 | III | None | *ermB* | *tetM* |  |
| DR3112 | 19 | CC19 | III | None |  |  |  |
| DR3116 | 23 | CC23 | Ia | I377V; V510I | *mef* | *tetM* |  |
| DR3118 | 1010 | CC452 | IV | I377V; V510I |  |  |  |
| DR3120 | 28 | CC19 | II | None | *mef* | *tetM* |  |
| DR3121 | 26 | CC26 | V | I377V |  | *tetM* |  |
| DR3122 | 144 | CC23 | Ia | I377V; V510I | *mef* | *tetM* |  |
| DR3123 | 17 | CC17 | III | I377V; G627V |  | *tetM* |  |
| DR3129 | 2114 | No CC | II | I377V; T617I | *lsaC* | *tetM* |  |
| DR3132 | 17 | CC17 | III | I377V; G627V |  | *tetM* |  |
| DR3138 | 19 | CC19 | V | None | *ermB; ermT* | *tetO* |  |
| DR3140 | 890 | CC452 | V | I377V; V510I | *ermB* | *tetM* |  |
| DR3143 | 17 | CC17 | III | G329V; I377V; G627V |  | *tetM* |  |
| DR3148 | 1 | CC1 | Ib | None |  | *tetM* |  |
| DR3150 | 1010 | CC452 | IV | I377V; V510I |  |  |  |
| DR3157 | 23 | CC23 | Ia | I377V; V510I | *mef* | *tetM* |  |
| DR3165 | 1010 | CC452 | IV | I377V; V510I |  |  |  |
| DR3170 | 1010 | CC452 | IV | I377V; V510I |  |  |  |
| DR3172 | 8 | CC12 | Ib | None |  | *tetM* |  |
| DR3173 | 144 | CC23 | Ia | I377V; V510I | *mef* | *tetM* |  |
| DR3180 | 28 | CC19 | II | None | *ermB* | *tetO* |  |
| DR3182 | 1 | CC1 | II | None |  | *tetM* |  |
| DR3201 | 498 | CC452 | V | I377V; V510I |  | *tetM* |  |
| DR3202 | 651 | No CC | III | I377V; T720S |  |  |  |
| DR3206 | 19 | CC19 | V | None | *ermB; ermT* | *tetO* |  |
| DR3215 | 498 | CC452 | Ib | I377V; V510I |  | *tetM* |  |
| DR3229 | 498 | CC452 | V | I377V; V510I |  | *tetM* |  |
| DR3230 | 17 | CC17 | III | I377V; G627V |  | *tetM* |  |
| DR3233 | 23 | CC23 | Ia | I377V; V510I |  | *tetM* |  |
| DR3234 | 498 | CC452 | Ib | I377V; V510I |  | *tetM* |  |
| DR3237 | 24 | CC452 | V | I377V; V510I |  | *tetM* |  |
| DR3241 | 651 | No CC | III | I377V; T720S |  |  |  |
| DR3248 | 17 | CC17 | III | I377V; G627V |  | *tetM* |  |
| DR3250 | 28 | CC19 | II | None |  | *tetM* |  |
| DR3259 | 23 | CC23 | Ia | I377V; V510I; E584K |  | *tetM* |  |
| DR3260 | 17 | CC17 | III | I377V; G627V |  | *tetM* |  |
| DR3268 | 2115 | No CC | II | I377V; T617I | *lnuB; lsaC; lsaE* | *tetM* |  |
| DR3273 | 651 | No CC | III | I377V; T720S | *ermB; lnuB; lsaE* |  |  |
| DR3274 | 1 | CC1 | II | None |  | *tetM* |  |
| DR3275 | 17 | CC17 | III | I377V; G627V |  | *tetM* |  |
| DR3276 | 24 | CC452 | II | I377V; V510I |  | *tetM* |  |
| DR3277 | 1010 | CC452 | IV | None |  |  |  |
| DR3294 | 347 | CC19 | II | None | *ermB* | *tetO* |  |
| DR3295 | 1 | CC1 | Ib | None |  | *tetM* |  |
| DR3297 | 347 | CC19 | II | None | *ermB* | *tetO* |  |
| DR3299 | 12 | CC12 | Ib | None |  | *tetM* |  |
| DR3305 | 498 | CC452 | V | I377V; V510I |  | *tetM* |  |
| DR3308 | 1 | CC1 | II | None |  | *tetM* |  |
| DR3310 | 860 | CC17 | III | G329V; I377V; G627V | *mef* | *tetM* |  |
| DR3313 | 1 | CC1 | II | None | *ermTR* | *tetM* |  |
| DR3318 | 17 | CC17 | III | G329V; I377V; G627V |  | *tetM* |  |
| DR3320 | 19 | CC19 | III | None |  | *tetM* |  |
| DR3322 | 1010 | CC452 | IV | I377V; V510I |  |  |  |
| DR3323 | 17 | CC17 | III | I377V; G627V |  | *tetM* |  |
| DR3328 | 498 | CC452 | Ib | I377V; V510I | *ermB* | *tetM; tetO* |  |
| DR3329 | 1010 | CC452 | IV | I377V; V510I |  |  |  |
| DR3333 | 1010 | CC452 | IV | I377V; V510I |  |  |  |
| DR3334 | 19 | CC19 | V | None | *ermB; ermT* | *tetO* |  |
| DR3338 | 19 | CC19 | V | None | *ermB; ermT* | *tetO* |  |
| DR3343 | 1010 | CC452 | IV | I377V; V510I |  |  |  |
| DR3344 | 23 | CC23 | Ia | I377V; V510I | *mef* | *tetM* |  |
| DR3345 | 1 | CC1 | Ib | None |  | *tetM* |  |
| DR3347 | 2116 | No CC | II | None | *ermB* | *tetO* |  |
