# Supplementary material for: Near-term pregnant women in the Dominican Republic experience high rates of Group B Streptococcus rectovaginal colonization with virulent strains
Source: PLOS Glob Public Health. 2023 Sep 21;3(9):e0002281. doi: 10.1371/journal.pgph.0002281 (PMC10513192; doi:10.1371/journal.pgph.0002281)
Supplement: Attachment — Submitted filename: Response to Reviewers.docx [file pgph.0002281.s005.docx]

PGPH-D-23-00911

Near-term pregnant women in the Dominican Republic experience high rates of Group B Streptococcus rectovaginal colonization with virulent strains

Response to Reviewers

22 July 2023

Dear Dr. Chaguza,

We wish to thank the editors and reviewers at *PLOS Global Public Health* for their thoughtful suggestions and for the opportunity to submit a revised manuscript. We are submitting a revised manuscript that incorporates these suggestions here. Please see our responses to each point in italicized text below.

Thank you again for considering our manuscript.

Sincerely,

Katherine Laycock, MD, DTM&H

Corresponding Author

Ryan White Center for Pediatric Infectious Diseases & Global Health

Indiana University School of Medicine

705 Riley Hospital Drive, Room 5919, Indianapolis, IN 46202, USA

Email: kalayco@iu.edu

Journal Requirements:

1. Please include a complete copy of PLOS’ questionnaire on inclusivity in global research in your revised manuscript. Our policy for research in this area aims to improve transparency in the reporting of research performed outside of researchers’ own country or community. The policy applies to researchers who have travelled to a different country to conduct research, research with Indigenous populations or their lands, and research on cultural artefacts. The questionnaire can also be requested at the journal’s discretion for any other submissions, even if these conditions are not met. Please find more information on the policy and a link to download a blank copy of the questionnaire here: https://journals.plos.org/globalpublichealth/s/best-practices-in-research-reporting. Please upload a completed version of your questionnaire as Supporting Information when you resubmit your manuscript.

**Response:** *Thank you for the journal’s commitment to inclusivity in global research. The Methods section of the text has been amended and the journal’s questionnaire has been uploaded to address the above.*

2. We noticed that you used "unpublished data" in the manuscript. We do not allow these references, as the PLOS data access policy requires that all data be either published with the manuscript or made available in a publicly accessible database. Please amend the supplementary material to include the referenced data or remove the references. Please review your reference list to ensure that it is complete and correct. If you have cited papers that have been retracted, please include the rationale for doing so in the manuscript text, or remove these references and replace them with relevant current references. Any changes to the reference list should be mentioned in the rebuttal letter that accompanies your revised manuscript. If you need to cite a retracted article, indicate the article’s retracted status in the References list and also include a citation and full reference for the retraction notice.

**Response:** *Thank you for these points. We have removed the reference to “unpublished data”. We have also revised the reference list to include three additional references, which support new text that was added in response to suggestions raised by the editor and reviewers below. The three additional references are as follows:*

*20. Metcalf BJ, Chochua S, Gertz RE, Hawkins PA, Ricaldi J, Li Z, et al. Short-read whole genome sequencing for determination of antimicrobial resistance mechanisms and capsular serotypes of current invasive Streptococcus agalactiae recovered in the USA. Clin Microbiol Infect. 2017 Aug;23(8):574.e7-574.e14.*

*21. Katz LS, Griswold T, Morrison SS, Caravas JA, Zhang S, den Bakker HC, et al. Mashtree: a rapid comparison of whole genome sequence files. J Open Source Softw. 2019 Dec 10;4(44).*

*22. Argimón S, Abudahab K, Goater RJE, Fedosejev A, Bhai J, Glasner C, et al. Microreact: visualizing and sharing data for genomic epidemiology and phylogeography. Microb Genom. 2016 Nov;2(11):e000093.*

Additional Editor Comments (if provided):

I suggest including antimicrobial resistance profiles based on the generated whole-genome sequencing data. Since the genomic data is already available, you could also optionally include a phylogenetic tree and describe the genomic diversity of the GBS isolates beyond the serotypes and MLST, for example, looking at the number of mutational (single nucleotide polymorphisms [SNP]) differences between isolates of the same ST/CC.

**Response:** *Thank you for this excellent suggestion. We have added a new table (S1 Table)*

*that includes isolate-level data for ST, CC, serotype, PBP2x mutations (relevant to beta-*

*lactam susceptibility), and resistance determinants for*

*macrolides/lincosamides/streptogramins, tetracyclines, and quinolone antibiotics. In*

*addition, we have provided relevant text about these data in the Methods and Results*

*sections. In addition, we have used the WGS data to generate a tree based on mash*

*(min-hash) distances using kmers. That tree, which allows comparison of mash vs. CC*

*groupings, is presented in Figure 2, and relevant details appear in the revised Methods*

*section. We elected not to pursue SNP-level analysis based on the absence of high-quality*

*reference genomes for many of the relevant CC/ST groupings.*

*While compiling the above new S1 Table and Figure 2, we noted a discrepancy in the CC*

*assignments presented in the originally submitted Figure 1. We apologize for this error*

*and have corrected the discrepancy in the newly submitted Figure 1.*

Reviewers' comments:

Reviewer's Responses to Questions

Comments to the Author

1. Does this manuscript meet PLOS Global Public Health’s publication criteria? Is the manuscript technically sound, and do the data support the conclusions? The manuscript must describe methodologically and ethically rigorous research with conclusions that are appropriately drawn based on the data presented.

Reviewer #1: Yes

Reviewer #2: Yes

2. Has the statistical analysis been performed appropriately and rigorously?

Reviewer #1: No

Reviewer #2: Yes

3. Have the authors made all data underlying the findings in their manuscript fully available (please refer to the Data Availability Statement at the start of the manuscript PDF file)? The PLOS Data policy requires authors to make all data underlying the findings described in their manuscript fully available without restriction, with rare exception. The data should be provided as part of the manuscript or its supporting information, or deposited to a public repository. For example, in addition to summary statistics, the data points behind means, medians and variance measures should be available. If there are restrictions on publicly sharing data—e.g. participant privacy or use of data from a third party—those must be specified.

Reviewer #1: Yes

Reviewer #2: Yes

4. Is the manuscript presented in an intelligible fashion and written in standard English? PLOS Global Public Health does not copyedit accepted manuscripts, so the language in submitted articles must be clear, correct, and unambiguous. Any typographical or grammatical errors should be corrected at revision, so please note any specific errors here.

Reviewer #1: Yes

Reviewer #2: Yes

5. Review Comments to the Author

Please use the space provided to explain your answers to the questions above. You may also include additional comments for the author, including concerns about dual publication, research ethics, or publication ethics. (Please upload your review as an attachment if it exceeds 20,000 characters)

**Reviewer #1:** In this manuscript, the authors seek to describe GBS prevalence among mothers seeking antenatal care at a healthcare facility in the Dominican Republic. They also describe the distribution of GBS clones circulating among this population to gauge the impact that GBS vaccines may have in preventing disease. This is a clear, concisely written, and important manuscript. I have only minor comments.

**Response:** *Thank you for your very helpful comments and suggestions. Please see our*

*responses to each point below.*

Line 127 – “Age bands” – please define this term.

**Response:** *We apologize for the ambiguity. We have revised the text to indicate that participants’ ages were recorded in pre-defined groups, i.e. ≤17 years, 18-24 years, 25-30 years, 31-35 years, 36-40 years, and >40 years.*

Line 154 – What do you mean by “contamination checks”? What threshold was used to determine contamination – for example, did you require that 100% of reads be classified as GBS to classify an isolate as “pure”?

**Response:** *We apologize for the ambiguity. Read classification using Kraken/Bracken is not perfect, so 100% of reads would not be expected to be classified as Streptococcus agalactiae/GBS even under ideal settings. We found that >88% of reads were classified as Streptococcus agalactiae/GBS in all samples, with most of the remaining reads being assigned to related streptococci, consistent with either lateral transfer events or ambiguity in assignment based on the presence of closely related genes. We have clarified this in the text.*

Line 162 – What is expected level of confidence and/or precision with a sample size of 350?

**Response:** *With a colonization rate of up to 35%, a sample size of 350 allows for a 5% margin of error at 95% confidence. We have clarified this in the revised text.*

Line 167: Please indicate in parentheses what methods of GBS detection were used, to remind readers.

**Response:** *Thank you for this suggestion. We have added the methods of GBS detection that were used (Carrot Broth^TM^, CHROMagar^TM^, or whole genome sequencing) to clarify this in the text.*

Line 169: Why didn’t you use t-tests to examine associations between continuous exposures and GBS prevalence, for example age, # of previous pregnancies, etc in Tables 1 and 2? You classification of some continuous variables into categories seems arbitrary for the purposes of statistical analysis.

**Response:** *Thank you for this comment. In order to reduce the amount of free text in the survey and limit errors in data collection, these variables were pre-specified as categories (e.g. age ≤17 years, 18-24 years, etc.; 0, 1, 2, 3, 4, 5, or >5 previous pregnancies) on the survey. Absolute numbers were not collected, and thus these variables could not be treated as continuous variables. In addition, the data for number of previous pregnancies were not normally distributed. For these reasons, we used chi-square analysis rather than t-test to examine the association between these variables and GBS colonization.*

Line 171: Mention p-value used to determine statistical significance.

**Response:** *A p-value <0.05 was considered significant. We have clarified this in the revised text.*

Line 241: “Four isolates had allele combinations that have not been previously described.” Are you referring to MLSTs here? If so, please state this. Also, you should assign STs for novel ST types and include these designations in parentheses here. This should be straightforward if you have WGS data.

**Response:** *We apologize for the ambiguous wording here. The reviewer is correct that we were referring to MLSTs when we said “allele combinations”. We have corrected the wording in the revised manuscript. In addition (though we did not specify this), we did submit the allele profiles to PubMLST, which assigned new MLST designations for the four strains (STs 2113-2116). These are provided in more detail in the new Table S1 that accompanies the revised manuscript.*

Line 268-270: Text appears to contrast what is reported in line 262, where you write that only GBS serotypes Ia, Ib, and II-V were observed in your cohort. How then are “serotypes III and II

... most prevalent” as reported in line 268?

**Response:** *The text “II-V” was meant to designate a range (i.e., serotypes II, III, IV, and V). We have clarified this in the revised text.*

Line 322 – Please qualify “very high.” What is the typical rate in other Latin American countries with more resources and/or greater access to maternal care?

**Response:** *Thank you for this point. We have expanded on this in the revised text. The measured incidence of early onset GBS disease among infants in the overall Latin America and Caribbean region is reported as 0.51 per 1000 live births, compared to 2.34 per 1000 live births in the DR. However, modeling estimates suggest that these numbers undercalculate the burden of neonatal disease.*

**Reviewer #2:** The article meets the criteria for PLOS Global public health journal publication and has a technically sound manuscript with conclusions that are supported by data presented in the results section. The mixed methods design for the primary research question posed undoubtedly projects an ethically and methodologically sound design with mentioned limitations and weaknesses.

This is further elaborated by the drawn conclusions which even with the mentioned biases in the design, not only answer the question posed but go on to identify the significance of the the hexavalent vaccine against the local group B streptococcus serotypes in the Dominican Republic. Despite the mentioned practical hindrances to implementing such a vital public health intervention in any developing nation, the conclusions also justify the development of better vaccines with more serotypes coverage through the recognition of 4 new and probably virulent group B streptococcus serotypes in a nation that might struggle to screen and prevent group b infections on top of the poor performances of the streptococcus isolation and diagnostic technology (carrot broth and Chromoagar).

An astounding attempt at statistical analysis of the data is seen through the sub-categorization of data captured from the interviews and attempt to find associations and correlations between the data but the mathematical formulas that were used to infer or defer any data correlation or association was just mentioned and not fully described. However, data availability seems adequate through data projected in the tables and the manuscript is presented in good palatable english.

A recommendation would be to provide the identification of 4 unrecognized species earlier in the results paragraph before mentioning the calculated sensitivity and specificity of the carrot-broth method of identifying and isolating the group b streptococcus. This is because of the logical presentation of the data in that paragraph which provides the numerical data to calculate sensitivity and specificity of the carrot-broth technique, but one would assume the provided figures, 87.1% and 96% to be wrong if you are working with a total of 350 eligible women who were tested yet 4 women had new strains that could not be classified such that the total tests included in the analysis became 346 instead. Otherwise this is an insightful research article that is relevant for its purpose and meets publication criteria.

**Response:** *Thank you for your very helpful comments and suggestions. We apologize for the ambiguity in our descriptions. As mentioned in the response to Reviewer #1, the four novel “allele combinations” that we mentioned were not meant to signify either new serotypes or species within GBS. These are new multilocus sequence types (MLSTs) of GBS. These new MLSTs have been assigned designations by PubMLST as listed above, but all four of these strains have serotypes (i.e., capsule types) that should be covered by the existing hexavalent vaccine candidate, and all should be detected using the carrot broth technique. We have attempted to clarify these issues in the revised text.*

6. PLOS authors have the option to publish the peer review history of their article (what does this mean?). If published, this will include your full peer review and any attached files.

Do you want your identity to be public for this peer review? If you choose “no”, your identity will remain anonymous but your review may still be made public.

For information about this choice, including consent withdrawal, please see our Privacy Policy.

Reviewer #1: No

Reviewer #2: Yes: Benson Tarisai Gombe
